# Association between estimated glucose disposal rate and atrial fibrillation recurrence in patients undergoing radiofrequency catheter ablation: a retrospective study

**DOI:** 10.1186/s40001-024-01911-7

**Published:** 2024-06-12

**Authors:** Xiaozhong Li, Zheng Zhou, Zhen Xia, Youzheng Dong, Si Chen, Fenfang Zhan, Zhichao Wang, Yang Chen, Jianhua Yu, Zirong Xia, Juxiang Li

**Affiliations:** 1 Department of Cardiovascular Medicine, the Second Affiliated Hospital, Jiangxi Medical College, Nanchang University, Nanchang, 330006 China; 2 Department of Anesthesiology, the Second Affiliated Hospital, Jiangxi Medical College, Nanchang University, Nanchang, 330006 China; 3 Department of Cardiovascular Medicine, the First Affiliated Hospital, Jiangxi Medical College, Nanchang University, Nanchang, 330006 China

**Keywords:** Estimated glucose disposal rate, Insulin resistance, Atrial fibrillation, Radiofrequency catheter ablation, Recurrence

## Abstract

**Objective:**

Previous studies have shown a clear link between insulin resistance (IR) and an elevated risk of atrial fibrillation (AF). However, the relationship between the estimated glucose disposal rate (eGDR), which serves as a marker for IR, and the risk of AF recurrence after radiofrequency catheter ablation (RFCA) remains uncertain. Therefore, this study aimed to examine the potential association between the eGDR and the risk of AF recurrence following RFCA.

**Methods:**

This retrospective study was conducted at Nanchang University Affiliated Second Hospital. The study enrolled 899 patients with AF who underwent RFCA between January 2015 and January 2022. The formula used to calculate the eGDR was as follows: 19.02 − (0.22 * body mass index) − (3.26 * hypertension) − (0.61 * HbA1c). Cox proportional hazard regression models and exposure–effect curves were used to explore the correlation between the baseline eGDR and AF recurrence. The ability of the eGDR to predict AF recurrence was evaluated using the area under the receiver operating characteristic curve (AUROC).

**Results:**

The study observed a median follow-up period of 11.63 months, during which 296 patients experienced AF recurrence. K‒M analyses revealed that the cumulative incidence AF recurrence rate was significantly greater in the group with the lowest eGDR (log-rank *p* < 0.01). Participants with an eGDR ≥ 8 mg/kg/min had a lower risk of AF recurrence than those with an eGDR < 4 mg/kg/min, with a hazard ratio (HR) of 0.28 [95% confidence interval (CI) 0.18, 0.42]. Additionally, restricted cubic spline analyses demonstrated a linear association between the eGDR and AF recurrence (*p* nonlinear = 0.70). The area under the curve (AUC) for predicting AF recurrence using the eGDR was 0.75.

**Conclusions:**

The study revealed that a decrease in the eGDR is associated with a greater AF recurrence risk after RFCA. Hence, the eGDR could be used as a novel biomarker for assessing AF recurrence risk.

## Introduction

Atrial fibrillation (AF) is the most prevalent cardiac arrhythmia and increases the risk of stroke, heart failure, and all-cause mortality [1]. According to the Global Burden of Disease Project [2], approximately 46.3 million people worldwide were affected by AF in 2016. This condition significantly impacts patients' quality of life and burdens public health. The primary treatment for patients with AF is radiofrequency catheter ablation (RFCA) [3]. However, despite its effectiveness, the rate of atrial arrhythmia remission remains unsatisfactory, and the success rates of a single procedure are suboptimal [4–6].

Identifying risk factors that affect sinus rhythm maintenance in patients with AF after RFCA is crucial due to the high rates of AF recurrence [7]. Recent studies have reported various systems and biomarkers to assess AF recurrence risk after RFCA [8, 9]. However, there is no consensus on a risk-scoring system or biomarkers for predicting rhythm outcomes after RFCA. Insulin resistance (IR) is an important risk factor for cardiovascular events [10] and is linked to atrial processes [11, 12]. The gold standard for evaluating IR is the euglycemic hyperinsulinemic clamp method [13], but its invasiveness and high cost make it unsuitable for widespread clinical implementation. The eGDR was calculated using waist circumference or body mass index (BMI), hypertension, and glycated hemoglobin A1c (HbA1c) to assess insulin resistance (IR) in individuals with type 1 diabetes [14]. Compared to the euglycemic hyperinsulinemic clamp method, eGDR is a reasonably accurate measure of IR and can be applied in clinical settings and large-scale research [15]. Several studies have suggested that lower eGDRs are associated with increased risks of acute ischemic stroke, all-cause mortality, and cardiovascular disease in both patients with type 1 and type 2 diabetes [14, 16]. However, there is currently limited evidence on the association between the eGDR and AF recurrence after RFCA. Therefore, our study aimed to evaluate this association.

## Methods

### Study design and population

This retrospective study included consecutive patients with AF who underwent their first RFCA procedure at the Second Affiliated Hospital of Nanchang University from January 2015 to January 2022. AF diagnosis was based on the absence of P waves and the presence of irregular F waves on the patient's electrocardiogram (ECG), with a frequency of 350–600 b.p.m. and an irregular ventricular response [17]. The exclusion criteria for this study were as follows: (1) AF induced by structural heart diseases and ischemia, such as myocardial infarction, decompensated heart failure, severe valvular heart disease, or rheumatic heart disease; and (2) reversible causes leading to AF, including acute thyrotoxicosis, pulmonary embolism, postoperative status, or solitary atrial flutter without AF. Contraindications to anticoagulation or left atrial thrombosis; (3) individuals with one or more history of ablation. The study’s protocol strictly adhered to the Helsinki Declaration and was approved by the ethics committee of the Second Affiliated Hospital of Nanchang University (2013) (No. 13, 2023, Nanchang, P. R. China).

### Data collection

General demographic and clinical information were obtained by reviewing the patient’s electronic medical records. The demographic data consisted of age, sex, BMI, systolic and diastolic blood pressure (BP), smoking status, alcohol status, AF type, duration of AF, RCFA strategy, medication status, New York Heart Association (NYHA) functional class, hypertension, coronary heart disease (CHD), heart failure (HF), diabetes mellitus, renal insufficiency, and dyslipidemia. The clinical information consisted of total cholesterol (TC), triglyceride (TG), high-density lipoprotein cholesterol (HDL-C), low-density lipoprotein cholesterol (LDL-C), estimated glomerular filtration rate (eGFR), glycated hemoglobin (HbA1C), uric acid (UA), brain natriuretic peptide (BNP), left atrial diameter (LAD), and left ventricle ejection fraction (LVEF) data.

### Definitions for the estimated glucose disposal rate and categorization

In this study, the formula eGDR = 19.02 − (0.22 * BMI) − (3.26 * hypertension) − (0.61 * HbA1c) was used to calculate the eGDR (mg/kg/min) as previously described [18].

BMI represents body mass index (kg/m^2^), hypertension is indicated as 1 for yes and 0 for no, and HbA1c represents HbA1c (DCCT %).

BMI was calculated by dividing weight (kg) by the square of height (m). Hypertension was defined as having a history of hypertension or treatment with antihypertensive medication and/or an SBP greater than 130 mm Hg and/or a DBP greater than 80 mm Hg [19]. High-performance liquid chromatography was used to measure HbA1c [20].

Per previous studies [18], the participants were categorized into four groups based on baseline eGDRs: < 4, 4–5.99, 6–7.99, and ≥ 8 mg/kg/min. The reference category was the lowest eGDR category (< 4 mg/kg/min).

### Radiometric catheter ablation strategy

Before RFCA, all patients were prescribed oral anticoagulants for a minimum of 30 days. At least five half-lives of antiarrhythmic drugs (AADs) were discontinued before RFCA. The procedure was conducted under local anesthesia. A decapolar catheter was inserted into the coronary sinus via the left subclavian vein, while a circumferential mapping catheter (Lasso, Biosense Webster, Diamond Bar, CA) was inserted into the pulmonary vein. Additionally, a 3.5-mm-diameter ablation electrode (Navistar Thermocool, Biosense Webster) was inserted into the left atrium (LA) through the right femoral vein. Continuous intravenous heparin was administered to maintain the activated coagulation time between 300 and 350 s. Initially, all patients underwent circumferential pulmonary vein isolation (PVI). For patients with paroxysmal AF, the ablation endpoint was the eradication of ectopic triggers and the inability to reinduce AF. In cases where non-PV triggers were present, further isolation was necessary for elimination. For instance, in patients exhibiting heightened signals or spontaneous ectopic activity originating from the superior vena cava (SVC), isolation of the SVC was performed. For patients with persistent AF, the ablation endpoint was the termination of AF and the restoration of sinus rhythm, and the ablation strategy involved PVI and substrate ablation (LA linear, complex fractionated atrial electrogram, and cavotricuspid isthmus ablation). After RFCA and AADs, oral anticoagulation was continued for three months.

### Follow-up

All patients underwent regular 12-lead ECG and 24-h Holter ECG at 1, 3, 6, 9, and 12 months during the first year after ablation during outpatient clinical follow-up. These tests were subsequently conducted every three months. The patients were also advised to promptly seek medical attention if they experienced any symptoms of AF recurrence, such as palpitations, dyspnea, fatigue, dizziness, chest pain, effort intolerance, or syncope. After three months, AADs were discontinued, but oral anticoagulation was continued according to the CHA2DS2-VASC scores. AF recurrence was defined as AF, atrial flutter, or atrial tachycardia lasting more than 30 s, based on ECG and Holter monitoring reports following a blanking period of three months [21].

### Statistical analysis

The continuous variables are presented as the mean ± standard deviation (SD). Categorical data are displayed as frequency percentages. Baseline characteristics across the eGDR < 4, 4–5.99, 6–7.99, and ≥ 8 mg/kg/min groups were compared using the Kruskal–Wallis test and Chi-squared test.

K‒M curves were used to estimate the rate of AF recurrence at follow-up for each eGDR group. Cox regression analysis was conducted to estimate the hazard ratios (HRs) and 95% confidence intervals (CIs) of eGDRs and AF recurrence while adjusting for potential confounding variables, including age, sex, duration of AF, UA, eGFR, BNP, AF type, LAD, LVEF, smoking status, alcohol consumption status, hyperlipidemia, CHD, HF, stroke, diabetes, and renal insufficiency. The dose–response relationship between the eGDR and AF recurrence was analyzed using restricted cubic splines.

Subgroup analyses of the eGDRs and AF recurrence rates were performed, and the HRs and 95% CIs for each subgroup are shown in forest plots. The predefined variables for subgroup analysis included sex, age (< 60 vs. ≥ 60 years), eGFR (< 90 vs. ≥ 90 ml/min/1.73 m2), LAD (< 40 vs. ≥ 40 mm), hyperlipidemia (yes vs. no), diabetes mellitus (yes vs. no), smoking status (yes vs. no), drinking status (yes vs. no), and AF type (paroxysmal vs. nonparoxysmal). The adjustment variables were duration of AF, CHD, HF, stroke, renal insufficiency, and LVEF. The eGDR ≥ 8 mg/kg/min group was compared with the eGDR < 4 mg/kg/min group in the subgroup analysis to enhance the statistical power. The predictive ability of various indicators for AF recurrence was evaluated using the area under the receiver operating characteristic curve (AUROC). Statistical significance was defined as a two-tailed *p* value of < 0.05. Data analyses were performed using R software version 4.1.3 (www.R-project.org) and SPSS software (version 20; IBM Corp., Armonk, NY, USA).

## Results

### Study population characteristics

**Fig. 1:**
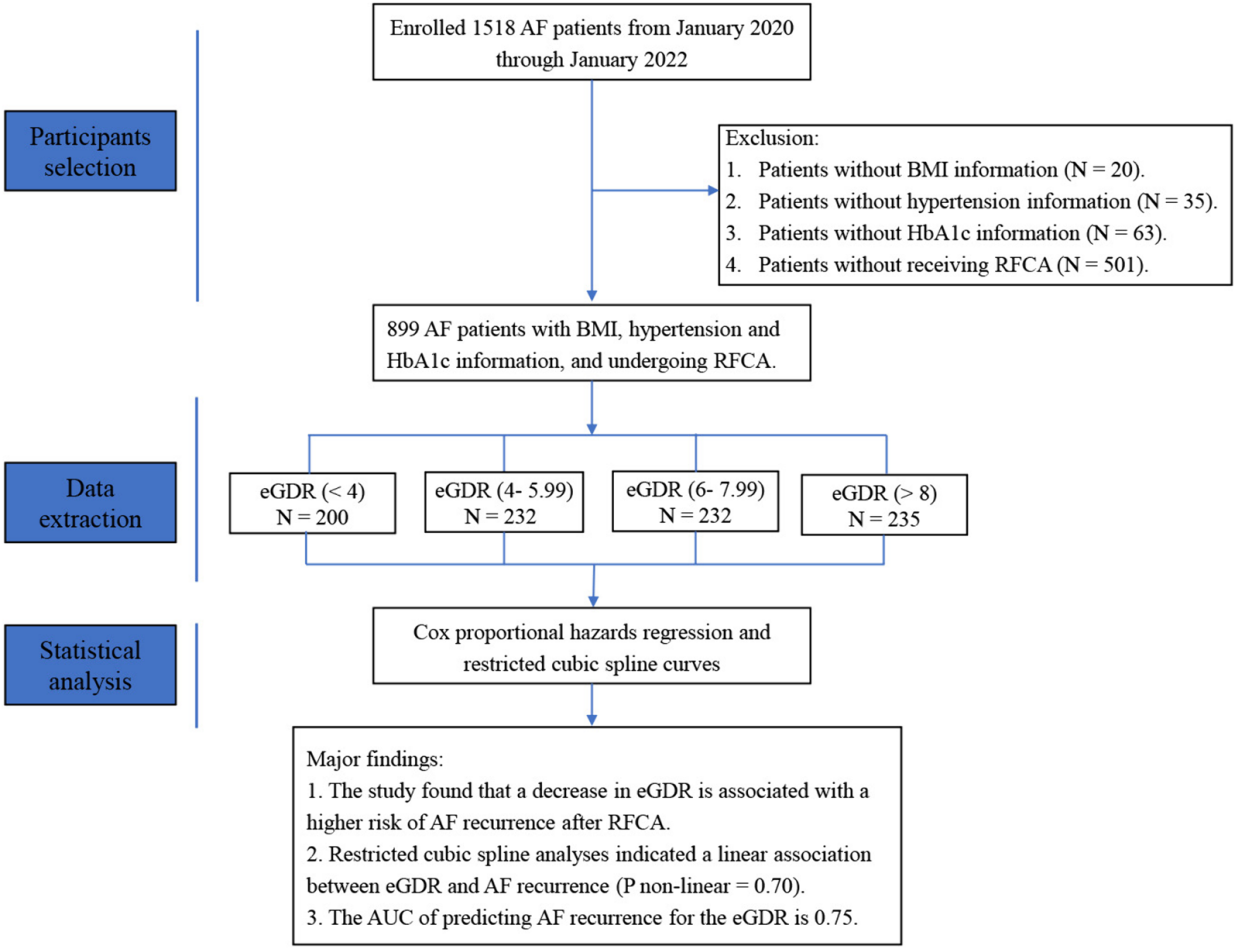
Flowchart of study selection. AF: atrial fibrillation; BMI: body mass index; HbA1c: hemoglobin A1c; RFCA: radiofrequency catheter ablation; AUC: area under the curve

A total of 1518 patients with AF who were hospitalized at Nanchang University Affiliated Second Hospital between January 20,215 and January 2022 were included in this study. Patients with missing BMI (*N* = 20), hypertension (*N* = 35), or HbA1c (*N* = 63) information were excluded. Patients who did not receive RFCA (*n* = 501) were also excluded. The final analysis included 899 patients (Fig. 1). In this study, 58.06% (522) of the included patients were female, and the average age was 64.45 (10.13) years. The median eGDR was 6.59 (2.13) mg/kg/min. Significant differences in baseline characteristics, including age, BMI, SBP, DBP, AF type, TG, HDL-c, glucose, HbA1c, eGFR, UA, LAD, NYHA functional class, addition ablation, hypertension, hyperlipidemia, diabetes, CHD, stroke, renal insufficiency, beta-blockers, CCB, lipid-lowering drugs, and AADs, were detected among the four subgroups (Table 1).

**Table 1:** Baseline characteristics by the tertiles of eGDR of the study population

Characteristics	Total	eGDR mg/kg/min				*p*
		< 4	4–5.99	6–7.99	≥ 8	
	899	200 (22.25%)	232 (25.81%)	232 (25.81%)	235 (26.14%)	
Age, years	64.45 (10.13)	65.60 (9.39)	67.00 (8.66)	63.00 (10.30)	62.40 (11.30)	< 0.01
Female, *n* (%)	522 (58.06)	114 (57.00)	125 (53.90)	155 (66.80)	128 (54.50)	0.02
BMI, kg/m^2^	24.67 (3.63)	27.70 (3.19)	23.80 (2.63)	25.60 (3.47)	21.90 (2.42)	< 0.01
SBP, mm Hg	131 (19)	136 (20)	131 (19)	130 (18)	127 (19)	< 0.01
DBP, mm Hg	77 (12)	80 (13)	79 (12)	77 (12)	74 (12)	< 0.01
Current smoking, *n* (%)	203 (22.60)	46 (23.00)	47 (20.30)	57 (24.60)	53 (22.60)	0.74
Current alcohol, *n* (%)	147 (16.35)	30 (15.00)	34 (14.70)	51 (22.00)	32 (13.60)	0.06
Paroxysmal	516 (57.40)	110 (55.00)	127 (54.70)	121 (52.20)	158 (67.20)	< 0.01
Duration of AF, months	34.38 (50.52)	32.30 (39.10)	31.80 (43.10)	38.60 (57.70)	34.30 (57.90)	0.47
Follow-up, months	13.78 (10.74)	14.40 (11.20)	14.40 (11.30)	13.50 (10.40)	12.90 (10.10/)	0.34
Laboratory results						
TC, mmol/L	4.35 ( 1.05)	4.44 (1.05)	4.26 (1.03)	4.34 (1.00)	4.38 (1.12)	0.33
TG,mmol/L	1.51 ( 0.97)	1.76 (0.89)	1.47 (0.91)	1.50 (1.16)	1.34 (0.83)	< 0.01
HDL-c,mmol/L	1.17 ( 0.32)	1.09 (0.27)	1.16 (0.31)	1.15 (0.32)	1.25 (0.35)	< 0.01
LDL-c, mmol/L	2.50 (0.81)	2.58 (0.79)	2.45 (0.80)	2.51 (0.79)	2.48 (0.84)	0.38
Glucose, mmol/L	5.29 (1.46)	5.81 (2.21)	5.34 (1.20)	5.15 (1.17)	4.94 (0.97)	< 0.01
HbA1c	8.62 (1.04)	9.96 (1.00)	7.97 (0.42)	8.49 (0.88)	8.24 (0.44)	< 0.01
eGFR, ml/min/1.73m^2^	84.19 (20.86)	82.3 (22.4)	78.3 (20.10)	87.5 (20.50)	88.4 (19.10)	< 0.01
UA, mmol/L	369 (109)	382 (111)	378 (119)	369 (98)	349 (104)	< 0.01
eGDR, mg/kg/min	6.59 (2.13)	3.69 (0.36)	5.49 (0.29)	7.40 (0.38)	9.35 (0.56)	< 0.01
BNP, pg/ml	287 (540)	244 (470)	296 (369)	279 (605)	323 (658)	0.49
LAD, mm	38.46 (7.09)	40.2 (5.54)	39.2 (5.60)	38.9 (5.86)	37.1 (5.79)	< 0.01
LVEF, (%)	59.99 ( 9.22)	60.0 (7.89)	60.6 (9.70)	60.1 (9.25)	59.2 (9.60)	0.47
NYHA functional class, *n* (%)						< 0.01
I	531 (59.07)	100 (50.00)	117 (50.40)	150 (64.70)	164 (69.80)	
II	286 (31.81)	83 (41.50)	96 (41.40)	62 (26.70)	45 (19.10)	
III	68 (7.56)	16 (8.00)	14 (6.03)	14 (6.03)	24 (10.2)	
IV	14 (1.56)	1 (0.50)	5 (2.16)	6 (2.59)	2 (0.85)	
Radiofrequency ablation strategy, *n* (%)						
PV isolation	899 (100)	200 (22.25)	232 (25.81)	232 (25.81)	235 (26.14)	1.00
SVC isolation	168 (18.69)	41 (20.50)	45 (19.40)	49 (21.10)	33 (14.00)	0.20
LA CFAE ablation	138 (15.35%)	37 (18.50)	35 (15.10)	40 (17.20)	26 (11.10)	0.14
LA linear ablation	390 (43.38%)	96 (48.00)	96 (41.40)	113 (48.70)	85 (36.20)	0.02
CTI ablation	273 (30.37)	72 (36.00)	69 (29.70)	71 (30.60)	61 (26.00)	0.16
Chronic disease, *n* (%)						
Hypertension	461 (51.28)	198 (99.00)	226 (97.40)	37 (15.90)	0 (0.00)	< 0.01
Hyperlipidemia	205 (22.80)	71 (35.50)	47 (20.30)	44 (19.00)	43 (18.30)	< 0.01
Diabetes	188 (20.91)	80 (40.00)	44 (19.00)	47 (20.30)	17 (7.23)	< 0.01
CHD	173 (19.24)	63 (31.50)	47 (20.30)	39 (16.80)	24 (10.20)	< 0.01
HF	171 (19.02)	41 (20.50)	55 (23.70)	39 (16.80)	36 (15.30)	0.09
Stroke	149 (16.57)	46 (23.00)	47 (20.30)	25 (10.80)	31 (13.20)	< 0.01
Renal insufficiency	203 (22.58)	25 (12.50)	32 (13.80)	14 (6.030)	12 (5.110)	< 0.01
Medication, *n* (%)						
Beta-blockers	356 (39.60)	95 (47.5%)	111 (47.8%)	82 (35.3%)	68 (28.9%)	< 0.01
CCB	204 (22.69)	70 (35.0%)	69 (29.7%)	37 (15.9%)	28 (11.9%)	< 0.01
Lipid-lowering drugs	375 (41.71)	121 (60.5%)	109 (47.0%)	85 (36.6%)	60 (25.5%)	< 0.01
MRA	105 (11.68)	25 (12.5%)	36 (15.5%)	20 (8.62%)	24 (10.2%)	0.11
Diuretics	133 (14.79)	31 (15.5%)	39 (16.8%)	31 (13.4%)	32 (13.6%)	0.69
Digoxin	79 (8.79)	15 (7.50%)	21 (9.05%)	18 (7.76%)	25 (10.6%)	0.63
Anticoagulation						0.72
Warfarin	33 (3.67)	7 (3.50%)	10 (4.31%)	9 (3.88%)	7 (2.98%)	
Dabigatran	564 (62.74)	131 (65.5%)	135 (58.2%)	155 (66.8%)	143 (60.9%)	
Rivaroxaban	276 (30.70)	55 (27.5%)	94 (31.44)	63 (27.2%)	78 (33.2%)	
AADs						0.01
Amiodarone	642 (71.41)	152 (76.00)	164 (70.70)	171 (73.70)	155 (66.00)	
Propafenone	78 (8.68)	16 (8.00)	20 (8.62)	18 (7.76)	24 (10.20)	
Dronedarone	7 (0.78)	0 (0.00)	1 (0.43)	2 (0.86)	4 (1.700)	
Sotalol	7 (0.78)	1 (0.50)	4 (1.72)	4 (1.72)	0 (0.00)	

The continuous data are expressed as mean (SD), or median (IQR); and the categorical data are present as numbers (percentages)

BMI: body mass index; AF: atrial fibrillation; SBP: systolic blood pressure; DBP: diastolic blood pressure; HbA1c: glycated hemoglobin; TG: triglycerides; TC: total cholesterol; LDL-C: lower-density lipoprotein cholesterol; HDL-C: high-density lipoprotein cholesterol; UA: uric acid; eGFR: estimated glomerular filtration rate; eGDR: estimated glucose disposal rate; BNP: brain natriuretic peptide; LAD: left atrial diameter; LVEF: left ventricle ejection fraction; PV: pulmonary vein; SVC: superior vena cava; LA: left atrium; CFAE: complex fractionated atrial electrogram; CTI: cavotricuspid isthmus; NYHA: New York Heart Association; CHD: coronary heart disease; HF: heart failure; CCB: calcium channel blockers; MRA: mineralocorticoid receptor antagonist; AADs: antiarrhythmic drugs

### Sinus rhythm maintenance rate curve

**Fig. 2:**
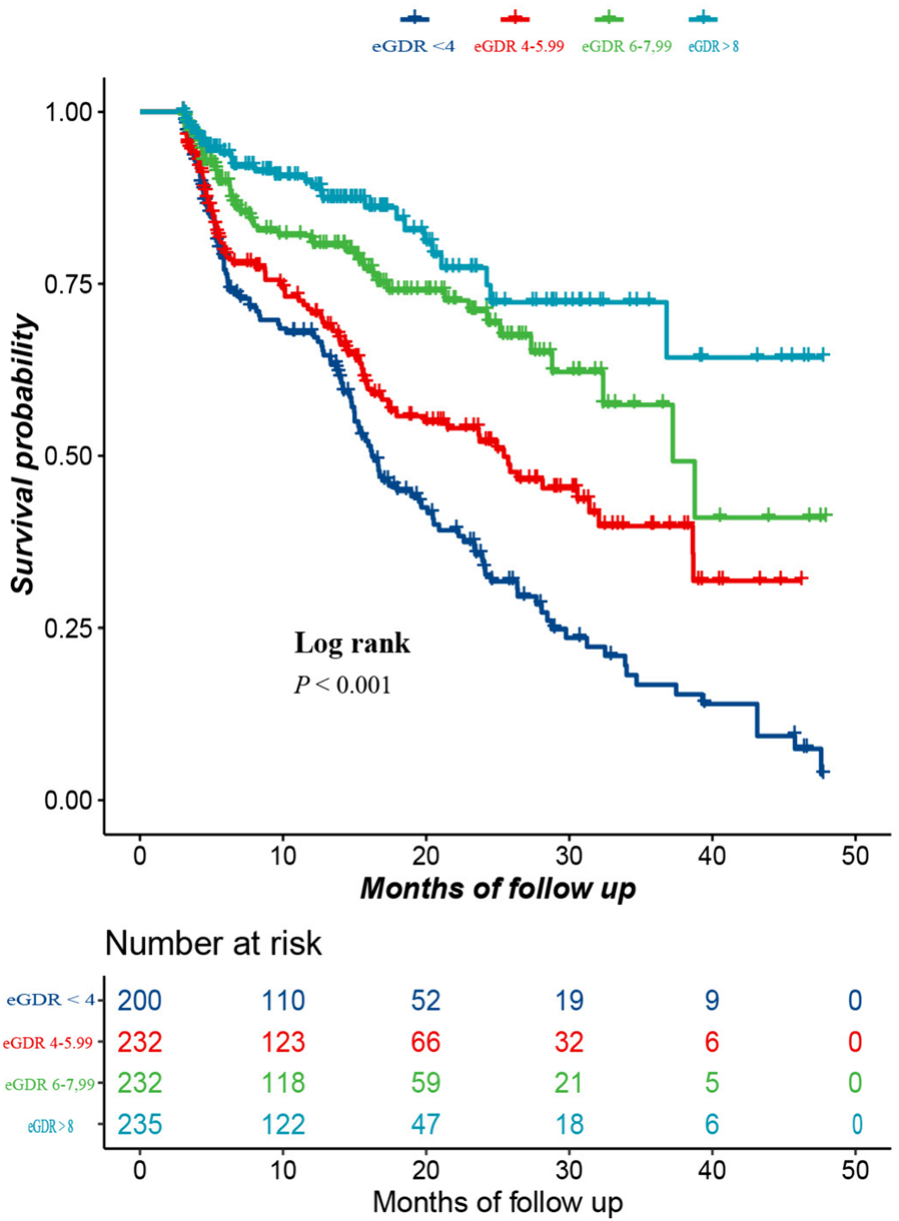
Sinus rhythm maintenance rate curve. eGDR: estimated glucose disposal rate

The Kaplan‒Meier analysis of AF recurrence based on baseline eGDRs (< 4, 4–5.99, 6–7.99, and ≥ 8 mg/kg/min) is shown in Fig. 2. Participants in the eGDR 4–5.99, 6–7.99, and ≥ 8 mg/kg/min groups had a significantly greater rate of sinus rhythm maintenance than did those in the eGDR < 4 mg/kg/min group (all log-rank *p* < 0.001).

### Association of the eGDR with the risk of AF recurrence

**Fig. 3:**
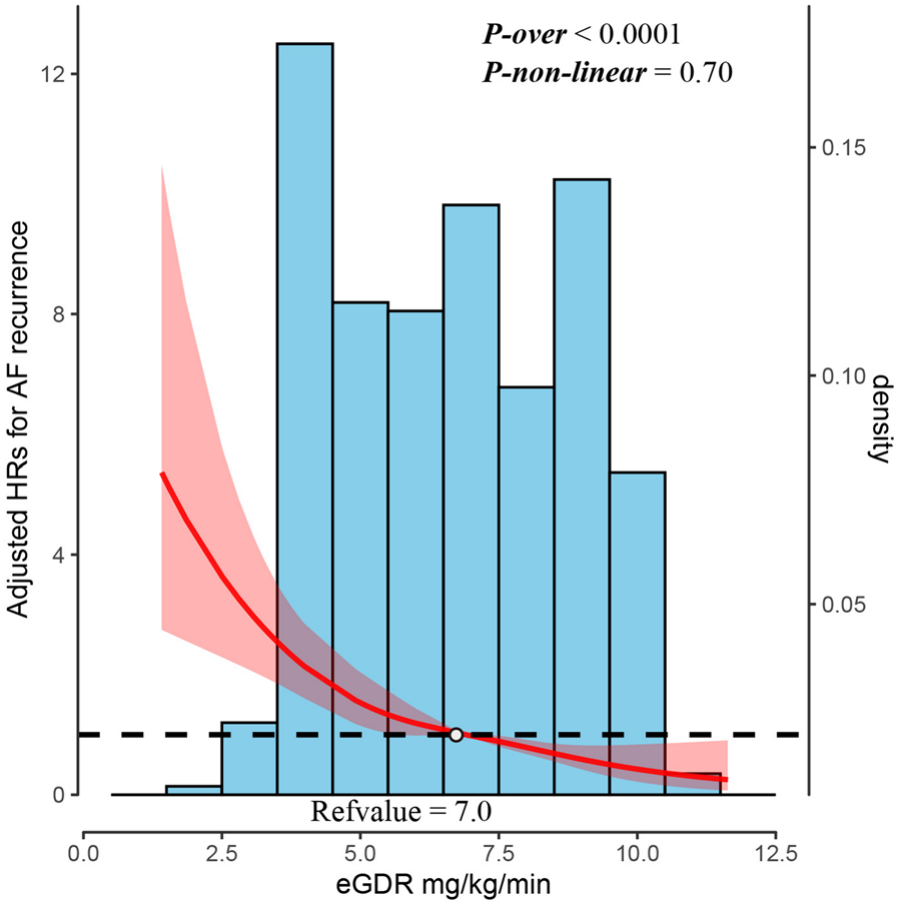
Hazard ratio and histogram of the probability distribution for AF recurrence according to the eGDR. The red curve with a light black dotted line indicates an adjusted odds ratio with 95% CI for AF recurrence according to an eGFR of 7.0 mg/kg/min. There were three knots for the cubic spline curves in the model. The adjustment factors included age, sex, duration of AF, AF type, eGFR, smoking status, alcohol consumption status, hyperlipidemia status, diabetes status, CHD status, HF status, stroke status, renal insufficiency status, LAD status, and LVEF. 95% CI 95% confidence interval; HR: hazard ratio; eGFR: estimated glucose disposal rate; AF: atrial fibrillation; UA: uric acid; eGFR: estimated glomerular filtration rate; BNP, brain natriuretic peptide; CHD: coronary heart disease; HF: heart failure; LAD: left atrial diameter; LVEF: left ventricle ejection fraction

After a median follow-up of 11.63 months, 296 patients experienced AF recurrence (Table 2). The study revealed a significant association between the eGDR and AF recurrence risk. When the eGDR was considered a categorical variable, the crude model showed that individuals with eGDRs of 4–5.99, 6–7.99, and ≥ 8 mg/kg/min had a significantly lower AF recurrence risk than those with eGDRs < 4 mg/kg/min, with HRs of 0.65 (0.50, 0.86), 0.37 (0.27, 0.52), and 0.24 (0.16, 0.36), respectively (Table 2). According to the fully adjusted model, individuals with eGDRs of 4–5.99, 6–7.99, and ≥ 8 mg/kg/min also had a significantly lower AF recurrence risk than those with eGDRs < 4 mg/kg/min, with HRs of 0.69 (0.52, 0.92), 0.39 (0.28, 0.56), and 0.28 (0.18, 0.42), respectively (Table 2). Furthermore, treatment of the eGDR as a continuous variable was negatively associated with AF recurrence (HR: 0.58, 95% CI 0.50, 0.67) (Table 2). After adjusting for multiple variables, restricted cubic spline analyses revealed a linear association between the eGDR and AF recurrence (*p* nonlinear = 0.70) **(**Fig. 3).

**Table 2:** The associations of eGDR with atrial fibrillation recurrence

	Cases/sample size	Crude Model HR (95%CI)	*p*-value	Model I HR (95%CI)	*p*-value	Model II HR (95%CI)	*p*-value
Per 1 SD increase	296/899	0.56 (0.48,0.63)		0.57 (0.49,0.64)	< 0.01	0.58 (0.50,0.67)	< 0.01
Categorical							
eGDR < 4 mg/kg/min	124/200	Ref (1.00)	1.00	Ref (1.00)	1.00	Ref (1.00)	1.00
eGDR 4–5.99 mg/kg/min	92/232	0.65 (0.50,0.86)	< 0.01	0.69 (0.53,0.91)	< 0.01	0.69 (0.52,0.92)	< 0.01
eGDR 6–7.99 mg/kg/min	50/232	0.37 (0.27,0.52)	< 0.01	0.38 (0.27,0.53)	< 0.01	0.39 (0.28,0.56)	< 0.01
eGDR ≥ 8 mg/kg/min	30/235	0.24 (0.16,0.36)	< 0.01	0.26 (0.17,0.39)	< 0.01	0.28 (0.18,0.42)	< 0.01
* p* trend		< 0.01		< 0.01		< 0.01	

Crude model: unadjusted any factor

Model I: multi‐factor model adjusted for age, sex, duration of AF, UA, eGFR, BNP, AF type, LAD, LVEF

Model II: multi‐factor model adjusted for Model I and smoking status, alcohol drinking status, hyperlipidemia, CHD, HF, stroke, diabetes, and renal insufficiency

95% CI 95% confidence interval; HR: hazard ratio; Ref: reference; eGDR: estimated glucose disposal rate; UA: uric acid; eGFR: estimated glomerular filtration rate; BNP, brain natriuretic peptide; CHD: coronary heart disease; HF: heart failure; LAD: left atrial diameter; LVEF: left ventricle ejection fraction

### Subgroup and sensitivity analysis

**Fig. 4:**
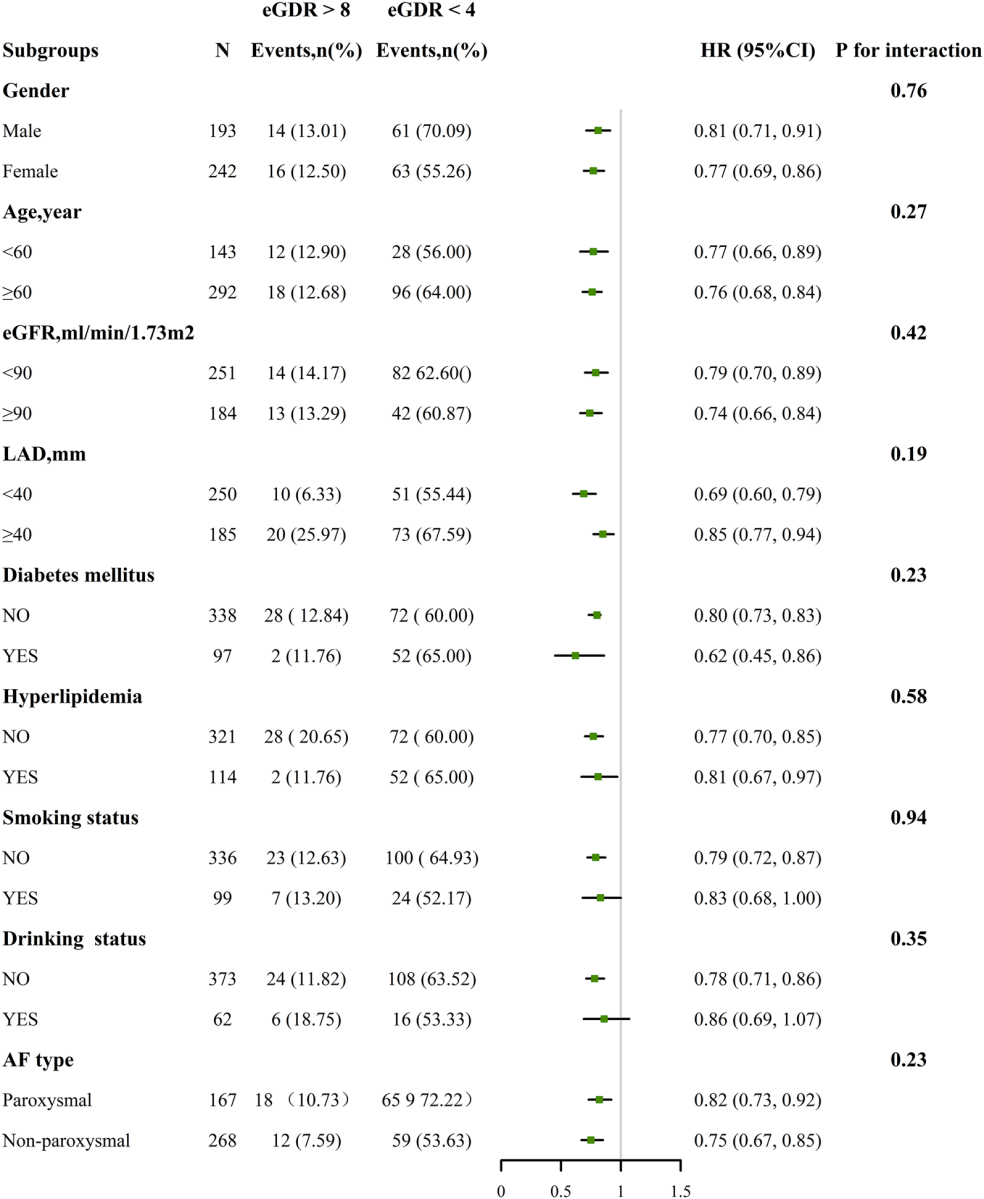
Associations between the eGDR (< 4 vs. ≥ 8 mg/kg/min) and AF recurrence in various subgroups. The results were adjusted for age, sex, duration of AF, UA, eGFR, BNP, AF type, LAD, LVEF, smoking status, alcohol consumption status, hyperlipidemia, CHD, HF, stroke, diabetes, and renal insufficiency if the above variables were not adjusted. 95% CI 95% confidence interval; HR: hazard ratio; eGDR: estimated glucose disposal rate; AF: atrial fibrillation; UA: uric acid; eGFR: estimated glomerular filtration rate; BNP, brain natriuretic peptide; CHD: coronary heart disease; HF: heart failure; LAD: left atrial diameter; LVEF: left ventricle ejection fraction

Subgroup analyses were conducted based on sex, age, eGFR, LAD, hyperlipidemia status, diabetes mellitus status, smoking status, drinking status, and AF type. Regarding AF recurrence, the stratified analyses did not reveal any significant interactions between the variables of interest and eGDR levels (all *p* interactions > 0.1) (Fig. 4).

### Receiver operating characteristic curve for the prediction of AF recurrence

**Fig. 5:**
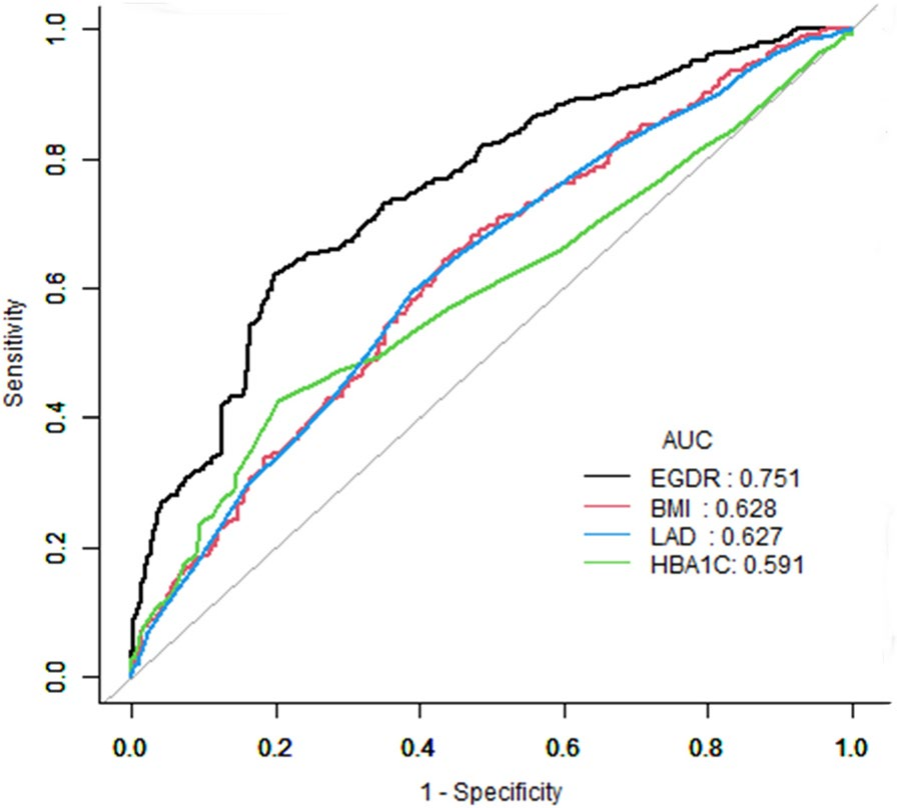
Receiver operating characteristic (ROC) curves and corresponding areas under the curve (AUCs). eGFR: estimated glucose disposal rate; AF: atrial fibrillation; BMI: body mass index; HbA1c: glycated hemoglobin; LAD: left atrial diameter

Figure 5 shows the ROC curves for the eGDR, BMI, HbA1c, and LAD in predicting AF recurrence. The ROC curve was used to determine the optimal cutoff point at which the maximal sensitivity and specificity were achieved. The areas under the curve (AUCs) for predicting AF recurrence for the eGDR, BMI, HbA1c, and LAD were 0.75 (95% CI 0.72, 0.79), 0.63 (95% CI 0.59, 0.67), 0.59 (95% CI 0.55, 0.63), and 0.62 (95% CI 0.58, 0.66), respectively. The sensitivity values were 33.17%, 56.55%, 79.60%, and 61.30%, respectively. The specificity values were 87.34%, 76.46%, 73.85%, and 75.1%, respectively. The optimal cutoff values for GDR, BMI, HbA1c, and LAD were 8.62, 24.50, 8.95, and 39.50, respectively (Table 3).

**Table 3:** Areas under the ROC curves for each parameter of the eGDR, BMI, HbA1c, and LAD for predicting atrial fibrillation recurrence

Parameters	Cut-off	Sensitivity	Specificity	AUC	95%CI	*p*-value
eGDR	8.62	33.17	87.34	0.75	0.72,0.79	< 0.01
BMI	24.50	56.55	76.46	0.63	0.59,0.67	< 0.01
HbA1c	8.95	79.60	73.85	0.59	0.55,0.63	< 0.01
LAD	39.50	61.30	75.1	0.62	0.58,0.66	< 0.01

95% CI 95% confidence interval; eGDR: estimated glucose disposal rate; BMI: body mass index; HbA1c: glycated hemoglobin; LAD: left atrial diameter

## Discussion

This study presents new evidence on the association between eGDR and AF recurrence in patients who underwent ablation for AF. The findings indicate that a lower eGDR is significantly linked to a greater AF recurrence risk, showing a linear relationship. Subgroup and sensitivity analyses further strengthened the consistent association between the eGDR and AF recurrence. The eGDR demonstrated an AUC of 0.75 for predicting AF recurrence.

Currently, the recognized modifiable risk factors for AF include hypertension, obesity, smoking, diabetes, and obstructive sleep apnea [22]. IR is an important early stage common to type 2 diabetes and a hallmark of obesity and metabolic syndrome. Therefore, IR may be the main underlying cause for the association of these factors with incident AF.

The gold standard technique for identifying and quantifying IR is the euglycemic hyperinsulinemic clamp, which is labor-intensive, time-consuming, and invasive, making it impractical for clinical practice [23]. An alternative indicator for assessing IR is the homeostasis model assessment of insulin resistance (HOMA-IR), which is derived from fasting insulin and glucose levels [24]. However, due to differences in measurement methods across laboratories, ensuring the accuracy of insulin measurements is difficult. Therefore, there is a need for simpler and more reliable indicators to assess IR in nondiabetic patients in clinical settings. The eGDR is a marker of IR that has similar accuracy to that of the euglycemic hyperinsulinemic clamp and is suitable for clinical practice [15]. Williams et al. demonstrated that the eGDR is a marker for assessing IR in individuals with type 1 diabetes [25]. Additionally, a lower eGDR is associated with an increased risk of stroke and death among patients with type 2 diabetes [14]. In a prospective cohort study of 15,773 patients with type 2 diabetes, the lowest eGDR tertile was significantly associated with all-cause mortality, even after adjusting for confounders, including diabetic kidney disease, over a 7.4-year follow-up period [26].

Some studies have suggested that IR may not have a significant association with AF. For instance, a study conducted on 3023 nondiabetic individuals from the Framingham Heart Study revealed that IR was not significantly associated with incident AF. Among those with the highest HOMA-IR, the HR was 1.18 (95% CI 0.84–1.65) compared to individuals in the other three quartiles of HOMA-IR [27]. Similarly, another study by Garg et al. involving a population-based cohort of 3601 individuals without underlying diabetes revealed a nonsignificant association between IR and incident AF. The HRs for those with the highest HOMA-IR and the lowest Gutt insulin sensitivity index was 0.76 (95% CI 0.63–0.91) and 1.09 (95% CI 0.92–1.30), respectively [28].

However, several studies have indicated a positive relationship between IR and AF. In the ARIC cohort, which consisted of 11,851 participants with a mean age of 54.0 years and 55.6% female, the lowest TyG index category (TyG < 8.80) and the highest TyG index category (TyG > 9.20) showed increased risks of AF compared to the middle TyG index category in a fully adjusted model. Specifically, the lowest TyG index category had an HR of 1.15 (95% CI 1.02, 1.29), while the highest TyG index category had an HR of 1.18 (95% CI 1.03, 1.37) [29]. Additionally, a retrospective observational study involving 356 patients reported a positive association between the TyG index and AF, with an OR of 2.09 (95% CI 1.41–3.10) [30]. Due to the unclear relationship between IR and AF, in this study, we aimed to explore the association between eGDR, a marker of IR, and AF recurrence.

Our findings provide evidence supporting a strong correlation between AF recurrence and IR, as evaluated through the eGDR. Subgroup analysis based on diabetes status revealed significant associations among both diabetic and nondiabetic participants. These results are consistent with prior research, such as a study of 232 patients who underwent ablation and were monitored for one year, which revealed that the homeostatic model assessment of insulin resistance (HOMA-IR) score was linked to AF recurrence (HR = 1.26, 95% CI 1.09–1.46) after adjusting for traditional risk factors [31]. Additionally, a study involving 2242 patients with AF who underwent RFCA demonstrated that individuals in the highest TYG index group had a greater risk of AF recurrence than did those in the lowest TYG index group (HR = 1.25, 95% CI 1.03–1.51) [21].

IR is a pathological condition in which cells or peripheral tissues fail to respond normally to insulin, leading to an inability to maintain glucose homeostasis in the body [32]. LA remodeling is a significant factor in the development of AF substrates [33].

Activation of the mTOR–S6K1 pathway and reduced insulin metabolic signaling have been linked to cardiac fibrosis [34]. Advanced glycation end products (AGEs) are formed in large quantities through protein glycation reactions triggered by hyperglycemia and glucotoxicity. AGEs stimulate inflammatory responses by binding to the cell surface receptor for AGEs, which in turn promotes fibrosis through the mitogen-activated protein kinase and Janus kinase signaling pathways. Additionally, AGEs contribute to the generation of reactive oxygen species (ROS), further exacerbating inflammation and fibrosis [35]. There is a correlation between IR and LA remodeling, even before diabetes onset [36]. IR contributes to increased LA size and impaired left ventricular diastolic function, both of which increase AF risk [27, 36]. IR may disrupt intracellular calcium homeostasis [32], induce oxidative stress [37], and lead to atrial interstitial fibrosis [38], thereby promoting LA remodeling. Additionally, IR may cause interatrial conduction delay and the formation of low-voltage areas [39], which can heighten susceptibility to AF. Furthermore, IR slows the left atrial conduction velocity, promotes re-entry, exacerbates atrial electrical remodeling, and increases the likelihood of AF recurrence after ablation [31, 40].

Catheter ablation is the preferred initial treatment for AF [41]. However, the rate of AF recurrence is approximately 30–50% after the first PV isolation [42]. A meta-analysis included 19 studies with a total of 6167 patients who underwent ablation, with a mean follow-up time of over 24 months. The results indicated that the single-procedure success rates for freedom from atrial arrhythmia were 53.1% (95% CI 46.2–60.0%) and 54.1% (95% CI 44.4% to 63.4%) for paroxysmal AF patients and 41.8% (95% CI 25.2% to 60.5%) for nonparoxysmal AF patients. Patients who underwent multiple procedures had a long-term success rate of 79.8% (95% CI 75.0% to 83.8%) at over three years follow-up [43]. In a randomized controlled trial, 589 patients with persistent atrial fibrillation were divided at a 1:4:4 ratio to three different treatments: ablation with PV isolation alone (67 patients), PV isolation plus ablation of electrograms showing complex fractionated activity (263 patients), or PV isolation plus additional linear ablation across the left atrial roof and mitral valve isthmus (259 patients). After 18 months, 59% of patients in the PV isolation alone group, 49% in the PV isolation plus complex electrogram ablation group, and 46% in the PV isolation plus linear ablation group were free from recurrent AF [44]. The current energy sources utilized in catheter ablation procedures include traditional radiofrequency, cryoballoons, and laser balloons. A randomized trial with 762 AF patients undergoing catheter ablation allocated 378 to cryoballoon ablation and 384 to radiofrequency ablation. After a 1.5-year follow-up, the risk of AF recurrence in the cryoballoon ablation group was comparable to that in the radiofrequency ablation group (HR: 0.96, 95% CI 0.76–1.22), and the safety profiles were also similar (HR: 0.78, 95% CI 0.52–1) [45]. In a study by Schmidt B. et al., 134 AF patients underwent catheter ablation, with 68 undergoing laser balloon ablation and 66 undergoing radiofrequency ablations. The AF-free rates after laser balloon ablation and radiofrequency ablation were 71.2% and 69.3%, respectively (*p* = 0.40) [46]. An observational study with two groups of 110 patients who had atrial fibrillation (AF) ablation with either a laser balloon ablation (55) or a cryoballoon ablation (55). At 12 months, the AF recurrence rates after laser balloon ablation were 30.9% and 29.1%, respectively (*p* = 0.54) [47]. As a result, the different energy sources used for catheter ablation do not affect the efficacy of AF ablation. Currently, several risk prediction scores are utilized to predict AF recurrence. However, these scores only offer moderate predictability [48]. Consequently, there is a demand for simpler and more easily accessible markers that can be employed in clinical settings to identify AF recurrence in ablation patients. The eGDR is rapid, feasible, and reliable, making it suitable for clinical application. Thus, the eGDR has the potential to serve as a reliable marker for AF recurrence.

## Limitations

While this study presents interesting findings, it is important to acknowledge its limitations. First, as this was a retrospective study, we were unable to establish a cause-and-effect relationship. Second, the study did not utilize the hyperinsulinemic-euglycemic clamp, the gold standard for evaluating IR. Nonetheless, there is a strong correlation between the hyperinsulinemic-euglycemic clamp and the eGDR [49]. Third, some patients with AF recurrence may be asymptomatic, leading to inaccuracies in the AF recurrence rate. Additionally, this study utilized 12-lead ECG and Holter monitoring instead of patch-type ECG, patient-triggered detection devices, or implantable loop recorders, which could have underestimated the AF recurrence rate. Fourth, although we adjusted for potential confounders as much as possible, there could be remaining unmeasured confounders influencing the observed associations. Fifth, it is worth noting that our subjects were exclusively from southeastern China; thus, the generalizability of the study results to individuals of different ethnicity backgrounds is uncertain. Therefore, further research involving diverse ethnicity populations is necessary.

## Conclusion

Our findings suggest that a lower eGDR is associated with AF recurrence after ablation. However, further large-scale observational studies are needed to validate these findings. Nevertheless, it is reasonable to consider the eGDR as a potential biomarker for predicting AF recurrence after ablation.

## Data Availability

The corresponding author will provide the raw data supporting the conclusions of this article without any hesitation or reservation.

## Abbreviations

AF: Atrial fibrillation
RFCA: Radiofrequency catheter ablation
eGDR: Estimated glucose disposal rate
ROC: Receiver operating characteristic
BMI: Body mass index
AF: Atrial fibrillation
HR: Hazard ratio
95% CI: 95% Confidence interval
HbA1c: Hemoglobin A1c
IR: Insulin resistance
ECG: Electrocardiograph
NYHA: New York Heart Association
BP: Blood pressure
HDL-C: High-density lipoprotein cholesterol
CHD: Coronary heart disease
HF: Heart failure
TC: Total cholesterol
TG: Triglyceride
HDL-C: High-density lipoprotein cholesterol
UA: Uric acid
LDL-C: Low-density lipoprotein cholesterol
BNP: Brain natriuretic peptide
LAD: Left atrial diameter
LVEF: Left ventricle ejection fraction
eGFR: Estimated glomerular filtration rate
AADs: Antiarrhythmic drugs
PVI: Pulmonary vein isolation
PV: Pulmonary vein
SVC: Superior vena cava
LA: Left atrium
SD: Standard deviation
AUROC: Receiver operating characteristic curve
HOMA-IR: Homeostatic model assessment for insulin resistance

## References

[1] Ding Y, Xu ZY, Liu HL (2019). Low deceleration capacity is associated with higher stroke risk in patients with paroxysmal atrial fibrillation. Chin Med J.

[2] Ma M, Zhi H, Yang S, Yu EY, Wang L (2022). Body mass index and the risk of atrial fibrillation: a mendelian randomization study. Nutrients.

[3] Chen X, Zhao J, Zhu K, Qin F, Liu H, Tao H (2021). The association between recurrence of atrial fibrillation and revascularization in patients with coronary artery disease after catheter ablation. Front Cardiovasc Med.

[4] Bertaglia E, Tondo C, De Simone A (2010). Does catheter ablation cure atrial fibrillation? Single-procedure outcome of drug-refractory atrial fibrillation ablation: a 6-year multicentre experience. Europace.

[5] Nilsson B, Chen X, Pehrson S, Køber L, Hilden J, Svendsen JH (2006). Recurrence of pulmonary vein conduction and atrial fibrillation after pulmonary vein isolation for atrial fibrillation: a randomized trial of the ostial versus the extraostial ablation strategy. Am Heart J.

[6] Pappone C, Rosanio S, Augello G (2003). Mortality, morbidity, and quality of life after circumferential pulmonary vein ablation for atrial fibrillation: outcomes from a controlled nonrandomized long-term study. J Am Coll Cardiol.

[7] Brandes A, Smit MD, Nguyen BO, Rienstra M, Van Gelder IC (2018). Risk factor management in atrial fibrillation. Arrhythm Electrophysiol Rev.

[8] Letsas KP, Efremidis M, Giannopoulos G (2014). CHADS2 and CHA2DS2-VASc scores as predictors of left atrial ablation outcomes for paroxysmal atrial fibrillation. Europace.

[9] Kosiuk J, Dinov B, Kornej J (2015). Prospective, multicenter validation of a clinical risk score for left atrial arrhythmogenic substrate based on voltage analysis: DR-FLASH score. Heart Rhythm.

[10] Laakso M, Kuusisto J (2014). Insulin resistance and hyperglycaemia in cardiovascular disease development. Nat Rev Endocrinol.

[11] Bohne LJ, Johnson D, Rose RA, Wilton SB, Gillis AM (2019). The association between diabetes mellitus and atrial fibrillation: clinical and mechanistic insights. Front Physiol.

[12] Wei Z, Zhu E, Ren C, Dai J, Li J, Lai Y (2021). Triglyceride-glucose index independently predicts new-onset atrial fibrillation after septal myectomy for hypertrophic obstructive cardiomyopathy beyond the traditional risk factors. Front Cardiovasc Med.

[13] DeFronzo RA, Tobin JD, Andres R (1979). Glucose clamp technique: a method for quantifying insulin secretion and resistance. Am J Physiol.

[14] Zabala A, Darsalia V, Lind M (2021). Estimated glucose disposal rate and risk of stroke and mortality in type 2 diabetes: a nationwide cohort study. Cardiovasc Diabetol.

[15] Komosinska-Vassev K, Gala O, Olczyk K, Jura-Półtorak A, Olczyk P (2020). The usefulness of diagnostic panels based on circulating adipocytokines/regulatory peptides, renal function tests, insulin resistance indicators and lipid-carbohydrate metabolism parameters in diagnosis and prognosis of type 2 diabetes mellitus with obesity. Biomolecules.

[16] Garofolo M, Gualdani E, Scarale MG (2020). Insulin resistance and risk of major vascular events and all-cause mortality in type 1 diabetes: a 10-year follow-up study. Diabetes Care.

[17] Davidson KW, Barry MJ, Mangione CM (2022). Screening for atrial fibrillation: US preventive services task force recommendation statement. JAMA.

[18] Linn W, Persson M, Rathsman B (2023). Estimated glucose disposal rate is associated with retinopathy and kidney disease in young people with type 1 diabetes: a nationwide observational study. Cardiovasc Diabetol.

[19] Whelton PK, Carey RM, Aronow WS (2018). 2017 ACC/AHA/AAPA/ABC/ACPM/AGS/APhA/ASH/ASPC/NMA/PCNA guideline for the prevention, detection, evaluation, and management of high blood pressure in adults: executive summary: a report of the American college of cardiology/American heart association task force on clinical practice guidelines. Hypertension.

[20] Lu J, He J, Li M (2019). Predictive value of fasting glucose, postload glucose, and hemoglobin A(1c) on risk of diabetes and complications in Chinese adults. Diabetes Care.

[21] Wang Z, He H, Xie Y (2024). Non-insulin-based insulin resistance indexes in predicting atrial fibrillation recurrence following ablation: a retrospective study. Cardiovasc Diabetol.

[22] Hindricks G, Potpara T, Dagres N (2021). 2020 ESC Guidelines for the diagnosis and management of atrial fibrillation developed in collaboration with the European Association for Cardio-Thoracic Surgery (EACTS): The Task Force for the diagnosis and management of atrial fibrillation of the European Society of Cardiology (ESC) Developed with the special contribution of the European Heart Rhythm Association (EHRA) of the ESC. Eur Heart J.

[23] Wang S, Shi J, Peng Y (2021). Stronger association of triglyceride glucose index than the HOMA-IR with arterial stiffness in patients with type 2 diabetes: a real-world single-centre study. Cardiovasc Diabetol.

[24] Zheng T, Ge B, Liu H (2018). Triglyceride-mediated influence of serum angiopoietin-like protein 8 on subclinical atherosclerosis in type 2 diabetic patients: results from the GDMD study in China. Cardiovasc Diabetol.

[25] Olson JC, Erbey JR, Williams KV (2002). Subclinical atherosclerosis and estimated glucose disposal rate as predictors of mortality in type 1 diabetes. Ann Epidemiol.

[26] Penno G, Solini A, Orsi E (2021). Insulin resistance, diabetic kidney disease, and all-cause mortality in individuals with type 2 diabetes: a prospective cohort study. BMC Med.

[27] Fontes JD, Lyass A, Massaro JM (2012). Insulin resistance and atrial fibrillation (from the Framingham Heart Study). Am J Cardiol.

[28] Garg PK, Biggs ML, Kaplan R, Kizer JR, Heckbert SR, Mukamal KJ (2018). Fasting and post-glucose load measures of insulin resistance and risk of incident atrial fibrillation: the cardiovascular health study. Nutr Metab Cardiovasc Dis.

[29] Liu X, Abudukeremu A, Jiang Y (2023). U-shaped association between the triglyceride-glucose index and atrial fibrillation incidence in a general population without known cardiovascular disease. Cardiovasc Diabetol.

[30] Chen S, Mei Q, Guo L (2022). Association between triglyceride-glucose index and atrial fibrillation: a retrospective observational study. Front Endocrinol.

[31] Wang Z, Wang YJ, Liu ZY (2022). Effect of insulin resistance on recurrence after radiofrequency catheter ablation in patients with atrial fibrillation. Cardiovasc Drugs Ther.

[32] Chan YH, Chang GJ, Lai YJ (2019). Atrial fibrillation and its arrhythmogenesis associated with insulin resistance. Cardiovasc Diabetol.

[33] Delgado V, Di Biase L, Leung M (2017). Structure and function of the left atrium and left atrial appendage: AF and stroke implications. J Am Coll Cardiol.

[34] Kim JA, Jang HJ, Martinez-Lemus LA, Sowers JR (2012). Activation of mTOR/p70S6 kinase by ANG II inhibits insulin-stimulated endothelial nitric oxide synthase and vasodilation. Am J Physiol Endocrinol Metab.

[35] Jia G, DeMarco VG, Sowers JR (2016). Insulin resistance and hyperinsulinaemia in diabetic cardiomyopathy. Nat Rev Endocrinol.

[36] Lee Y, Cha SJ, Park JH (2020). Association between insulin resistance and risk of atrial fibrillation in non-diabetics. Eur J Prev Cardiol.

[37] Korantzopoulos P, Kolettis TM, Galaris D, Goudevenos JA (2007). The role of oxidative stress in the pathogenesis and perpetuation of atrial fibrillation. Int J Cardiol.

[38] Maria Z, Campolo AR, Scherlag BJ, Ritchey JW, Lacombe VA (2020). Insulin treatment reduces susceptibility to atrial fibrillation in type 1 diabetic mice. Front Cardiovasc Med.

[39] Shigematsu Y, Hamada M, Nagai T (2011). Risk for atrial fibrillation in patients with hypertrophic cardiomyopathy: association with insulin resistance. J Cardiol.

[40] Hijioka N, Kamioka M, Matsumoto Y (2019). Clinical impact of insulin resistance on pulmonary vein isolation outcome in patients with paroxysmal atrial fibrillation. J Cardiovasc Electrophysiol.

[41] Cosedis Nielsen J, Johannessen A, Raatikainen P (2012). Radiofrequency ablation as initial therapy in paroxysmal atrial fibrillation. N Engl J Med.

[42] Tang Q, Guo XG, Sun Q, Ma J (2022). The pre-ablation triglyceride-glucose index predicts late recurrence of atrial fibrillation after radiofrequency ablation in non-diabetic adults. BMC Cardiovasc Disord.

[43] Ganesan AN, Shipp NJ, Brooks AG (2013). Long-term outcomes of catheter ablation of atrial fibrillation: a systematic review and meta-analysis. J Am Heart Assoc.

[44] Verma A, Jiang CY, Betts TR (2015). Approaches to catheter ablation for persistent atrial fibrillation. N Engl J Med.

[45] Kuck KH, Brugada J, Fürnkranz A (2016). Cryoballoon or radiofrequency ablation for paroxysmal atrial fibrillation. N Engl J Med.

[46] Schmidt B, Neuzil P, Luik A (2017). Laser balloon or wide-area circumferential irrigated radiofrequency ablation for persistent atrial fibrillation: a multicenter prospective randomized study. Circ Arrhythm Electrophysiol.

[47] Schiavone M, Gasperetti A, Montemerlo E (2022). Long-term comparisons of atrial fibrillation ablation outcomes with a cryoballoon or laser-balloon: a propensity-matched analysis based on continuous rhythm monitoring. Hellenic J Cardiol May-Jun.

[48] Kornej J, Schumacher K, Dinov B (2018). Prediction of electro-anatomical substrate and arrhythmia recurrences using APPLE, DR-FLASH and MB-LATER scores in patients with atrial fibrillation undergoing catheter ablation. Sci Rep.

[49] Williams KV, Erbey JR, Becker D, Arslanian S, Orchard TJ (2000). Can clinical factors estimate insulin resistance in type 1 diabetes?. Diabetes.

